# Advanced Imaging and Preoperative MR-Based Cinematic Rendering Reconstructions for Neoplasms in the Oral and Maxillofacial Region

**DOI:** 10.3390/diagnostics15010033

**Published:** 2024-12-26

**Authors:** Adib Al-Haj Husain, Milica Stojicevic, Nicolin Hainc, Bernd Stadlinger

**Affiliations:** 1 Clinic of Cranio-Maxillofacial and Oral Surgery, Center of Dental Medicine, University of Zurich, 8032 Zurich, Switzerland; milica.stojicevic@zzm.uzh.ch (M.S.); bernd.stadlinger@zzm.uzh.ch (B.S.); 2 Department of Cranio-Maxillofacial and Oral Surgery, University Hospital Zurich, University of Zurich, 8032 Zurich, Switzerland; 3 Department of Neuroradiology, Clinical Neuroscience Center, University Hospital Zurich, University of Zurich, 8091 Zurich, Switzerland; nicolin.hainc@usz.ch; 4 Department of Cranio-Maxillofacial Surgery, GROW School for Oncology and Reproduction, Maastricht University Medical Centre, 6229 Maastricht, The Netherlands

**Keywords:** cinematic rendering, image postprocessing, magnetic resonance imaging, oral and maxillofacial surgery, neoplasm

## Abstract

This case study highlights the use of cinematic rendering (CR) in preoperative planning for the excision of a cyst in the oral and maxillofacial region of a 60-year-old man. The patient presented with a firm, non-tender mass in the right cheek, clinically suspected to be an epidermoid cyst. Conventional imaging, including dental magnetic resonance imaging (MRI) protocols, confirmed the lesion’s size, location, and benign nature. CR reconstructions, combining advanced algorithms and novel skin presets, allow for the generation of highly realistic, three-dimensional visualizations from conventional imaging datasets. CR provided an enhanced, detailed depiction of the lesion within its anatomical context, significantly improving spatial understanding for surgical planning. The surgical excision was performed without complications, and histological analysis confirmed the diagnosis of a benign epidermoid cyst with no evidence of dysplasia or malignancy. This case demonstrates the potential of CR to refine preoperative planning, especially in complex anatomical regions such as the face and jaw, by offering superior visualization of superficial and deep structures. Thus, the integration of CR into clinical workflows has the potential to lead to improved diagnostic accuracy and better surgical outcomes.

**Figure 1:**
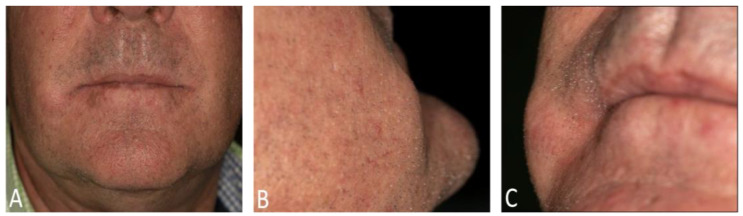
We present the case of a 60-year-old male patient, otherwise medically healthy, with a history of 80 pack-years of smoking and occasional alcohol consumption. He was referred to the Clinic of Cranio-Maxillofacial and Oral Surgery for further evaluation of a firm mass in the right cheek, initially discovered by his dentist. The patient reported no associated pain or discomfort, and there was no history of recent trauma or infection. On extraoral clinical examination, a firm, non-mobile, well-circumscribed, and non-tender lesion was palpated in the right buccal region. No evidence of pus or inflammation was observed. (**A**) frontal, (**B**) sagittal, and (**C**) lateral view of the initial presentation. The intraoral examination revealed the presence of bilateral linea alba and a whitish, non-wipable, hyperkeratotic mucosal lesion, which was non-tender. Furthermore, a whitish, non-removable coating was observed on the dorsal surface of the tongue. A clinical diagnosis of a leukokeratosis nicotina palati and a suspected epidermoid cyst (atheroma) in the right cheek was established. However, due to the lesion’s unclear nature, further imaging studies were recommended to evaluate its characteristics more accurately and to determine an appropriate treatment plan.

**Figure 2:**
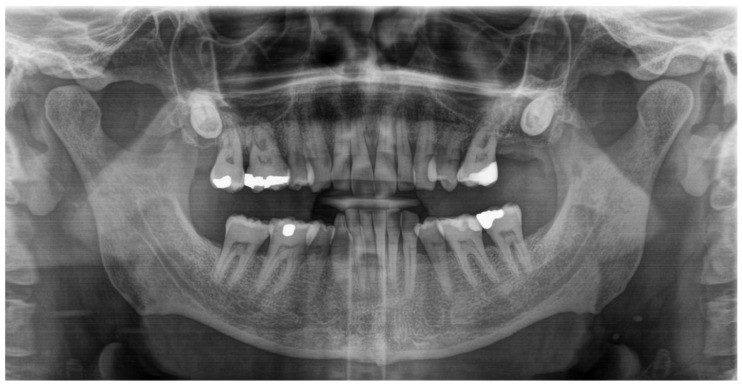
An orthopantomogram (OPG) was conducted as part of the X-ray-based diagnostic workup to evaluate the potential involvement of osseous structures within the jaws and surrounding anatomical regions that may have contributed to the lesion’s clinical presentation. The OPG revealed the absence of any underlying osseous pathologies or involvement of the mandible or maxilla, and did not reveal any pathological dental findings.

**Figure 3:**
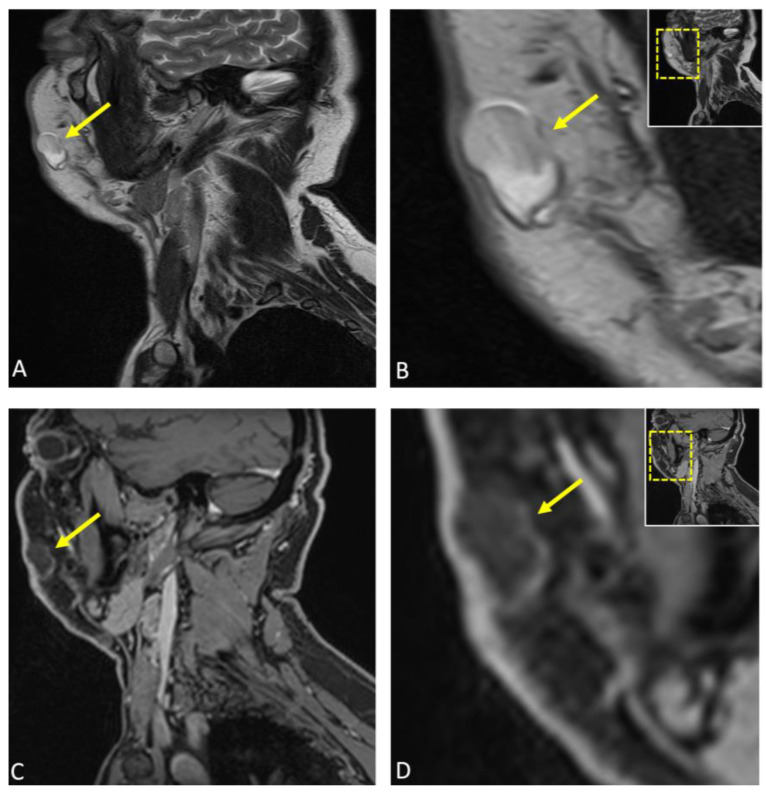
Subsequently, considering the patient’s age and medical history, a comprehensive diagnostic imaging workup was conducted, encompassing both conventional and dental MRI protocols for visualizing the facial skull and suprahyoidal neck using a contrast agent. This approach was undertaken with the objective of attaining a more detailed understanding of the patho-anatomy within the specified region, delineating soft tissues, and characterizing the lesion’s internal composition. The patient underwent MRI at 3 Tesla (MAGNETOM Skyra, release VE11E, Siemens Healthineers, Erlangen, Germany) with the following MRI protocols: Sagittal reconstructions of the lesion are presented in (**A**) T2-weighted turbo spin echo (TSE) and (**C**) T1-weighted Dixon MRI, with an enlarged view in (**B**,**D**), respectively. The lesion (arrow) measured 24 × 12 × 16 mm and was identified as a well-circumscribed macrocystic mass within the subcutaneous fat of the right cheek, specifically located in the superficial fascia. Mild compression of the levator anguli oris muscle was observed, with broad-based contact between the lesion and the overlying skin. No significant contrast enhancement was noted in the lesion’s capsule, although there was a slight increase in protein concentration and isotropic diffusion restriction, findings consistent with those observed in epidermoid cysts. Perilesional edema was not present. The rest of the examination revealed no abnormalities in the depicted anatomical areas.

**Figure 4:**
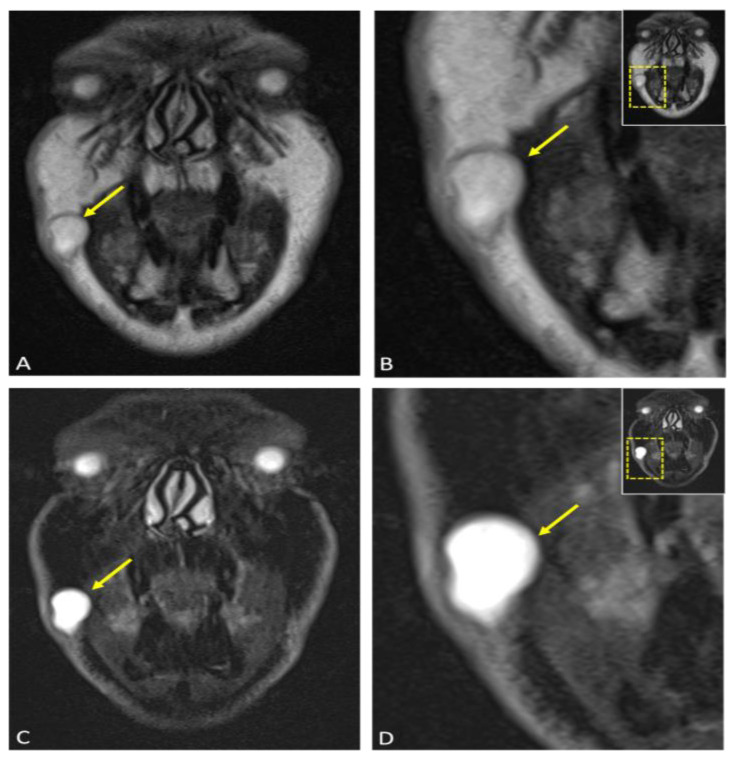
Coronal reconstructions of the lesion (arrow) are displayed in (**A**) T2-weighted Dixon MRI in-phase (water + fat) and (**C**) T2-weighted Dixon MRI water-only phase, which can be used as a fat-suppressed image. Enlarged views of the lesion are provided in (**B**,**D**) for greater clarity. The imaging findings are consistent with an epidermoid cyst, characterized by a well-circumscribed appearance and lack of infiltration into adjacent structures or inflammatory changes.

**Figure 5:**
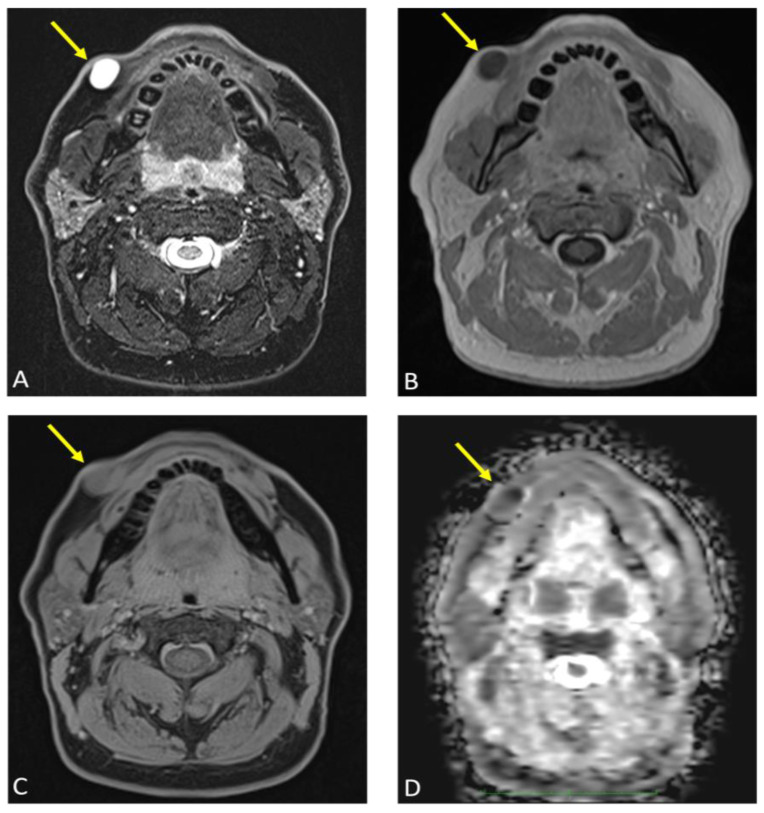
Axial reconstructions of the lesion (arrow) are displayed in multiple MR sequences: (**A**) T2-weighted turbo spin echo (TSE) Dixon, (**B**) T1-weighted volumetric interpolated breath-hold examination (VIBE) Dixon, (**C**) T1-weighted Star Volumetric Interpolated Breath-hold Examination (StarVIBE) Dixon technique, and (**D**) diffusion-weighted imaging (DWI). No significant contrast enhancement was noted in the lesion’s capsule, although there was a slight increase in protein concentration and isotropic diffusion restriction. These images collectively illustrate the lesion’s characteristics and contribute to the comprehensive assessment of its anatomical features and potential implications for the diagnosis and treatment of an epidermoid cyst.

**Figure 6:**
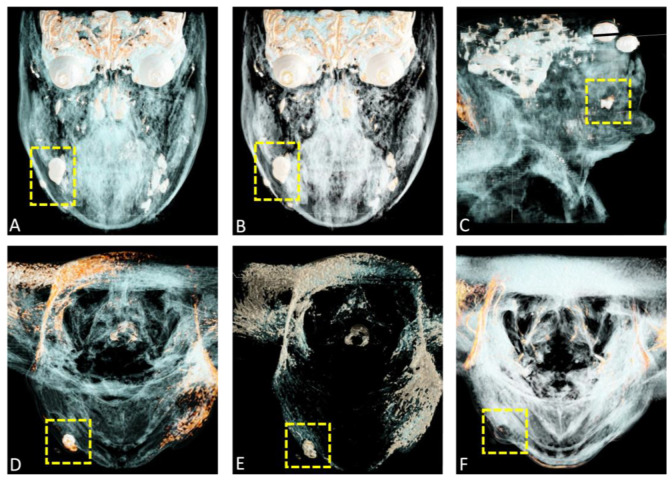
A complete surgical excision of the lesion was subsequently planned. Cinematic rendering (CR), which employs advanced algorithms to generate highly realistic three-dimensional visualizations from conventional imaging modalities like magnetic resonance imaging (MRI), enhances the representation of superficial structures by incorporating novel skin presets. Such a high level of detail is especially valuable in dermatosurgery, where precise characterization of lesions is essential for effective diagnosis and treatment planning. (**A**,**B**) present coronal views, while (**C**) displays a sagittal reconstruction, and (**D**–**F**) show axial preoperative CR reconstructions derived from MRI datasets. This innovative approach combines radiological diagnostics with cinematography, allowing surgeons to interact with three-dimensional volumetric models of the patient’s patho-anatomy. As a result, surgeons gain a clearer spatial understanding of lesion locations and potential surgical challenges. By employing advanced algorithms and novel skin presets, CR greatly enhances the visualization of superficial structures, offering detailed depictions of skin lesions within their anatomical context. Integrating CR into imaging workflows empowers surgeons to make informed decisions, particularly in complex anatomical regions such as the face and jaw, where precision is critical.

**Figure 7:**
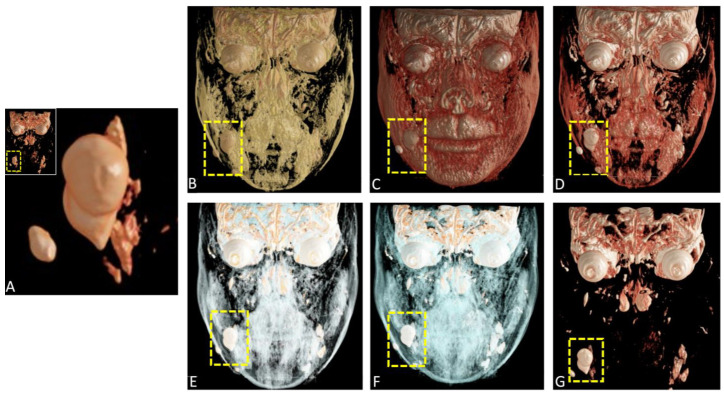
Preoperative coronal cinematic rendering (CR) reconstructions from magnetic resonance imaging (MRI) datasets using a variety of presets (**A**–**G**). The integration of various presets further optimizes the representation of superficial structures, contributing to improved diagnostic accuracy and aiding in surgical planning.

**Figure 8:**
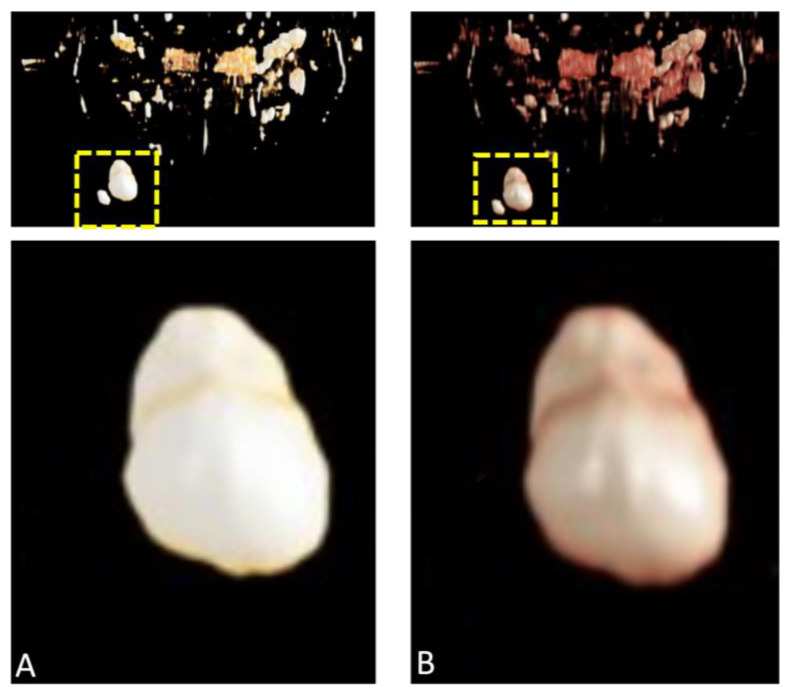
(**A**,**B**) Detailed segmentation of the neoplasia from magnetic resonance imaging (MRI) data was performed for precise surgical planning. The images, acquired using a dental-specific MRI protocol, allowed the segmentation of a well-defined lesion and reconstruction in three dimensions. This segmentation allowed precise localization and delineation of the lesion within the surrounding anatomical structures. The surgical procedure was conducted under local anesthesia in an aseptic environment with no perioperative complications. An incision was made at the optimal angle for accessing the right cheek lesion, followed by dissection using dissecting scissors. The neoplasia was fully excised, followed by coagulation and suturing with 5-0 PTFE material. The excised tissue was submitted for histological analysis, which confirmed the presence of a benign epidermoid cyst accompanied by a significant foreign body giant cell reaction and horn granulomas. There was no evidence of dysplasia, malignancy, or significant inflammatory infiltration. Recent advancements in medical imaging have markedly enhanced perioperative diagnostics for neoplasms in the oral and maxillofacial region. The broad spectrum of soft tissue tumors and tumor-like lesions, which range from benign, slow-growing masses to aggressive, malignant neoplasms with diverse etiologies, represents a considerable challenge in clinical decision-making [1,2]. Innovations such as novel detector technologies [3], optimized high-resolution MR sequences [4], and advanced post-processing applications [5] have enabled more accurate visualization of tumor boundaries, infiltration into surrounding tissues, and vascular involvement [6]. These technologies have the potential to enable more precise surgical planning, minimize complications, and provide individualized treatment strategies, which may ultimately result in improved patient outcomes and reduced recurrence rates. Furthermore, the incorporation of artificial intelligence and machine learning into imaging analysis has contributed to the early detection and characterization of neoplastic lesions [7]. Cutaneous cysts of the scalp are characterized as tumors or tumor-like lesions that present one or more cavities, either clinically or histologically. An epithelial capsule encloses true cysts, while pseudocysts are encapsulated by non-epithelialized walls composed of connective or granulation tissue. The contents of these cysts can vary widely in viscosity and consistency, ranging from calcified keratin to semi-solid or fluid material. Developmental cysts in the oral and maxillofacial region are typically congenital, clinically well-defined, and slow-growing. Additionally, they frequently exhibit connections to deeper structures. The classification of all these cysts is typically based on the histological characteristics of the cyst wall and the anatomical location [8]. Epidermoid cysts are one of the most common types of cysts in humans. These subcutaneous formations are primarily associated with areas of the body exhibiting sebaceous glands and hair follicles. These lesions are most frequently observed on the scalp, face, retroauricular region, trunk, and upper back. While they generally occur as solitary cysts, multiple cysts may also develop in close proximity. Epidermoid cysts are typically benign and slow-growing, making them harmless in most cases and often not requiring treatment [8]. However, the cyst wall can continuously produce keratin, leading to cyst enlargement and, in some instances, rupture. Although rupture itself is not inherently dangerous, it can trigger inflammation and/or infection, potentially leading to abscess formation, which can pose a significant health risk. In such cases, the cysts may fill with pus, resulting in an inflammatory response characterized by erythema and pain. However, epidermoid cysts can present a diagnostic challenge due to their overlapping imaging features with other benign and malignant lesions [9]. Given their high prevalence in facial skin, these lesions are among the benign lesions most frequently encountered by dermatologists and surgeons [8]. Consequently, precise localization and characterization are essential for effective surgical intervention and optimal patient outcomes, particularly in symptomatic, infected, or cosmetic indications. The field of dental magnetic resonance imaging (MRI) has witnessed a notable advancement with the introduction of novel MR protocols, including Black Bone MRI and CT-like MRI, in conjunction with the use of sophisticated coils [4]. These advancements provide surgeons with a comprehensive understanding of the patho-anatomy in the region of interest, thereby enabling improved perioperative radiological and surgical management [4,10]. This may potentially reduce the incidence of intraoperative complications and the risk of misdiagnosis. CR is an innovative and transformative technique, first introduced in 2016, that has gained attention for its ability to create highly realistic, three-dimensional visualizations from conventional imaging modalities, such as computed tomography (CT) and MRI [5]. Its implementation in the medical field is expanding rapidly, including in fields such as cardiology, musculoskeletal imaging, and head and neck imaging [11]. By merging radiological diagnostics with cinematography, CR offers significant improvements over traditional rendering methods, acting as a bridge between radiology and surgical planning. In this case, post-processing of the high-resolution DICOM MRI data was completed in under two minutes using the Cinematic Anatomy software (CA VB128, Siemens Healthineers AG, Erlangen, Germany) [12]. The workflow included manual adjustments to optimize visualization, which could be more time-intensive depending on the complexity of the case. The workup was conducted by an experienced oral and maxillofacial surgeon skilled in CR techniques, ensuring clinically meaningful and precise representations. CR enables surgeons to interact with lifelike, volumetric models of the patient’s anatomy, facilitating a better spatial understanding of lesion location and potential surgical challenges, particularly in regions with dense anatomical structures. In the context of neoplasms in the oral and maxillofacial region, CR has the potential to provide an unprecedented level of anatomical detail, offering clear depictions of both soft tissue and bony structures [13,14]. Therefore, integrating CR into imaging workflows enables surgeons to make more informed decisions, especially in complex anatomical regions like the face and jaw, where high precision is critical. As shown in this article, the use of CR is a valuable technique in the context of preoperative planning, particularly in cases involving superficial neoplasms in the oral and maxillofacial region. By integrating sophisticated algorithms with innovative skin presets, CR offers a three-dimensional, highly realistic representation of superficial structures, thereby enabling surgeons to gain a more precise understanding of lesion location, size, and anatomical relationships. This enhanced visualization has the potential to facilitate optimal surgical decision-making, minimizing inefficiencies and the risk associated with surgical procedures, which is particularly important in complex regions such as the head and neck.

## Data Availability

The datasets used and/or analyzed during the current study are available from the corresponding author upon request.

## References

[1] Błochowiak K., Farynowska J., Sokalski J., Wyganowska-Świątkowska M., Witmanowski H. (2019). Benign tumours and tumour-like lesions in the oral cavity: A retrospective analysis. Postep. Dermatol. Alergol..

[2] Peker E., Öğütlü F., Karaca İ., Gültekin E.S., Çakır M. (2016). A 5 year retrospective study of biopsied jaw lesions with the assessment of concordance between clinical and histopathological diagnoses. J. Oral. Maxillofac. Pathol..

[3] Sartoretti T., Wildberger J.E., Flohr T., Alkadhi H. (2023). Photon-counting detector CT: Early clinical experience review. Br. J. Radiol..

[4] Al-Haj Husain A., Sekerci E., Schönegg D., Bosshard F.A., Stadlinger B., Winklhofer S., Piccirelli M., Valdec S. (2022). Dental MRI of Oral Soft-Tissue Tumors-Optimized Use of Black Bone MRI Sequences and a 15-Channel Mandibular Coil. J. Imaging.

[5] Stadlinger B., Valdec S., Wacht L., Essig H., Winklhofer S. (2020). 3D-cinematic rendering for dental and maxillofacial imaging. Dentomaxillofac. Radiol..

[6] Vishwanath V., Jafarieh S., Rembielak A. (2020). The role of imaging in head and neck cancer: An overview of different imaging modalities in primary diagnosis and staging of the disease. J. Contemp. Brachyther..

[7] Mahmood H., Shaban M., Rajpoot N., Khurram S.A. (2021). Artificial Intelligence-based methods in head and neck cancer diagnosis: An overview. Br. J. Cancer.

[8] Kim C.S., Na Y.C., Yun C.S., Huh W.H., Lim B.R. (2020). Epidermoid cyst: A single-center review of 432 cases. Arch. Craniofac. Surg..

[9] Chung C.M., Wee S.J., Lim H., Cho S.H., Lee J.W. (2020). Skin malignancy initially misdiagnosed as a benign epidermal cyst. Arch. Craniofac. Surg..

[10] Al-Haj Husain A., Schmidt V., Valdec S., Stadlinger B., Winklhofer S., Schönegg D., Sommer S., Özcan M., Al-Haj Husain N., Piccirelli M. (2023). MR-orthopantomography in operative dentistry and oral and maxillofacial surgery: A proof of concept study. Sci. Rep..

[11] Brookmeyer C., Chu L.C., Rowe S.P., Fishman E.K. (2024). Clinical implementation of cinematic rendering. Curr. Probl. Diagn. Radiol..

[12] Comaniciu D., Engel K., Georgescu B., Mansi T. (2016). Shaping the future through innovations: From medical imaging to precision medicine. Med. Image Anal..

[13] Steffen T., Winklhofer S., Starz F., Wiedemeier D., Ahmadli U., Stadlinger B. (2022). Three-dimensional perception of cinematic rendering versus conventional volume rendering using CT and CBCT data of the facial skeleton. Ann. Anat..

[14] Huellner M.W., Engel K., Morand G.B., Stadlinger B. (2024). Cinematic rendering of [18F]FDG-PET/MR. Eur. J. Nucl. Med. Mol. Imaging.

